# Selection of Macrolide and Non-Macrolide Resistance with Mass Azithromycin Distribution: A Community-Randomized Trial

**DOI:** 10.1056/NEJMoa2002606

**Published:** 2020-11-12

**Authors:** Thuy Doan, Lee Worden, Armin Hinterwirth, Ahmed M. Arzika, Ramatou Maliki, Amza Abdou, Lina Zhong, Cindi Chen, Catherine Cook, Elodie Lebas, Kieran S. O’Brien, Catherine E. Oldenburg, Eric D. Chow, Travis C. Porco, Marc Lipsitch, Jeremy D. Keenan, Thomas M. Lietman

**Affiliations:** 1 Francis I Proctor Foundation, University of California San Francisco, USA; 2 Department of Ophthalmology, University of California San Francisco, USA; 3 The Carter Center, Niger; 4 Ministry of Health, Niger; 5 Programme National de Santé Oculaire, Niger; 6 Department of Biochemistry and Biophysics, University of California San Francisco, USA; 7 Department of Epidemiology and Biostatistics, University of California San Francisco, USA; 8 Department of Epidemiology, Harvard T.H. Chan School of Public Health, Harvard University, MA, USA; 9 Institute for Global Health Sciences, University of California San Francisco, USA

## Abstract

**Background:**

Biannual mass azithromycin distributions to preschool children for 2 years have been shown to reduce childhood mortality in sub-Saharan Africa, but at the cost of amplifying macrolide resistance. Here we investigated the gut resistome of children after 4 additional biannual distributions were given.

**Methods:**

In the Niger site of the MORDOR (*Macrolides Oraux pour Réduire les Décès avec un Oeil sur la Résistance*) trial, 30 villages were enrolled in a sister trial in which they were randomized to mass distribution of either azithromycin or placebo every 6 months for 4 years, with treatments offered to all children 1 to 59 months of age. Rectal samples were collected at baseline, 36 months, and 48 months for gut resistome analysis. All field and laboratory personnel were masked to the participants’ original assignments. The primary outcome was the ratio in macrolide resistance determinants between treatment arms at 48 months.

**Results:**

Over the entire 48-month period, mean (±SD) drug coverage was 86.6±12% in the placebo villages and 83.2±16.4% in the azithromycin villages. Macrolide resistance determinants were more common in the azithromycin arm compared to the placebo arm at 36 months (7.4-fold difference, 95% confidence interval 4.0 to 17.9-fold) and at 48 months (7.5-fold difference, 95% CI: 4.0 to 21.7-fold). Continued mass azithromycin distributions also selected for non-macrolide resistance determinants, including beta-lactams, the antibiotic class prescribed most frequently in this region.

**Conclusions:**

The study revealed that repeated mass azithromycin distributions may propagate antibiotic resistance. (ClinicalTrials.gov, NCT02047981)

## INTRODUCTION

Globally, 5.4 million children under 5 years of age died in 2017, with the highest rates of childhood mortality occurring in sub-Saharan Africa^1^. Biannual mass distributions of oral azithromycin to 1-59 month-old children reduced childhood mortality by 18% over 2 years in Niger, suggesting that this simple intervention could be a promising strategy for combatting childhood mortality^2,3^. The same intervention, however, resulted in an increase in the prevalence of macrolide resistance in *Streptococcus pneumoniae* colonizing the nasopharynx, as well as an increase in genetic macrolide resistance determinants in the gut of children who lived in the azithromycin-treated communities^4,5^. Resistance to non-macrolide antibiotics was not observed after 4 rounds of biannual azithromycin distributions in Niger^4,5^.

The emergence of antibiotic resistance observed after 2 years of treatment calls into question the long-term effectiveness of such an intervention to improve childhood mortality and its potential contribution to the growing global burden of antibiotic resistance. In this study, we evaluated the effects of longer-term biannual mass azithromycin distributions on the gut resistome, a reservoir of antimicrobial resistance genes in the body^6,7^.

## METHODS

**Ethical Review:** We obtained ethical approval for the study from the University of California, San Francisco (UCSF) Committee for Human Research and the Ethical Committee of the Niger Ministry of Health. The study was undertaken in accordance with the Declaration of Helsinki. We obtained verbal informed consents from guardians of children prior to treatment and swab collection given the low literacy rate in Niger.

**Study Design:** An ancillary cluster-randomized trial was initiated in the MORDOR study area of Niger in November 2013, concurrent with the main MORDOR trial.^8^ A group of 30 communities was randomly selected from the larger pool of communities in the main MORDOR trial and randomized in a 1:1 ratio to the same interventions implemented in MORDOR: biannual mass treatment of 1-59 month-old children with either azithromycin or placebo. Changes in antibiotic resistance determinants were the prespecified outcomes, assessed at annual monitoring visits.

**Setting:** The study took place in the Loga and Boboye departments of Niger from November 2013 until May 2019. Only non-urban communities were included.

**Participants:** The randomization unit was the *grappe*, which is the smallest government health unit in Niger. Grappes, termed villages or communities for the present report, were eligible for inclusion if the most recent government census documented a population between 200 and 2,000 inhabitants. All children aged 1 to 59 months and weighing at least 3800 grams were eligible for treatment. One village declined participation after undergoing 4 rounds of treatment.

**Randomization and Masking:** Randomization and interventions were performed at the community level. The trial biostatistician generated the randomization sequence using R software, version 3.5.1 (R Foundation for Statistical Computing). Allocation concealment was achieved by offering the treatment to all children in the community. Study drug was labelled with one of 6 letters (i.e., 3 for azithromycin and 3 for placebo) but otherwise the packaging and appearance of study drug was identical in the two arms. All field workers, study coordinators, investigators (except for the biostatistician), and laboratory personnel were masked to the link between the letters and the treatment assignments.

**Intervention:** All children 1 to 59 months old were identified in biannual censuses. Trained personnel directly observed study drug being taken by participating children. Single-dose oral azithromycin suspension (height-based dosing to a target dose of ≥20 mg/kg) or placebo suspension was offered at months 0, 6, 12, 18, 24, 30, 36, and 42. Children known to be allergic to macrolides were not treated.

**Sample Collection:** 50 children (or all children if less than 40 in that community) were randomly selected from the census for the monitoring visits at months 0, 36, and 48, with the goal of sampling 40 children per village. Separate random samples were selected at each monitoring visit; individual children were not followed longitudinally. Children could be born into and aged out of eligibility. The baseline visit took place before any treatments were distributed, the 36-month visit occurred approximately 6 months after the sixth round of treatment, and 48-month visit took place approximately 6 months following the eighth round of treatment. A flocked rectal swab (FLOQSwab) was inserted approximately 2 cm into the anus of each child and twisted 180 degrees, then removed and stored in DNA/RNA Shield (Zymo Research). A new pair of gloves was worn for each study participant. The samples were placed on ice in the field, stored in a -20°C freezer in Niger, then shipped to UCSF, where they were stored at -80°C until processing.

**Metagenomic DNA Sequencing:** Up to 40 total rectal samples from each village were pooled for sequencing; if more than 40 samples were collected from a community a simple random sample of 40 was chosen and processed ^9^. Thus, a total of 67 collected samples were not processed. A total of 3,232 rectal samples were processed, yielding 30 pooled samples at baseline, 29 pooled samples each at 36 and 48 months. Each pool contained 500 uL of each of the rectal samples from a village. DNA was extracted from 350 uL of each pooled sample using the Norgen stool DNA isolation kit (Norgen) per manufacturer’s instructions. The DNA concentration of each pooled sample was quantified using the Qubit® DNA HS Assay Kit (ThermoFisher Scientific) and normalized to 5ng/uL for sequencing library preparation. 5 uL of the pooled DNA was used to prepare DNA libraries using the New England Biolabs’ (NEB) NEBNext Ultra II DNA Library Prep Kit and then amplified with 10 PCR cycles. Library size and concentration were determined using the High Sensitivity DNA Chips (Agilent Technologies) and the Qubit® DNA HS Assay Kit (ThermoFisher Scientific), respectively. Libraries were then pooled and sequenced on the Illumina NovaSeq 6000 using 150-nucleotide (nt) paired-end sequencing.

**Assessment of Resistance Gene Determinants:** All paired-end reads were subjected to three rounds of human sequencing read removal. In an initial removal step, all paired-end reads were aligned to the human reference genome 38 (hg38) and the Pantroglodytes genome (panTro4, 2011, UCSC), using the Spliced Transcripts Alignment to a Reference (STAR) aligner (v2.5.4b) ^10^. Unaligned reads were quality filtered using PriceSeqFilter (v1.2) with the “-rnf 90” and “-rqf 85 0.98” settings^11^. Reads that were at least 95% identical were compressed by cd-hit-dup (cd-hit v4.7) ^12^. Furthermore, read pairs with a compression score less than 0.45 using the Lempel-Ziv-Welch algorithm were discarded because of low complexity^13^. Another round of human reads removal was performed using the very-sensitive-local mode of Bowtie2 (v2.3.4.1) with the same hg38 and panTro4 reference genomes described above. Lastly, the remaining reads were subject to taxonomic classification using the Centrifuge Taxonomic Classifier engine (v.1.0.3-beta), using an index created from the NCBI nucleotide non-redundant sequences (3/3/2018)^14^. Any reads classified under NCBI taxonomic IDs 7711 (Chordata), 6340 (Annelida), 6656 (Arthropoda), 2157 (Archaea), 33090 (Viridiplantae), and 81077 (artificial sequences) were also removed.

Non-host reads were then aligned to the MEGARes reference antimicrobial database (version 1.0.1) using the Burrows-Wheeler Aligner (BWA) with default settings^15^. Only antibiotic resistance determinants with gene fraction of >80% were identified as present in the sample and included for further analyses^4,5,16^. Each identified antibiotic resistance determinant was classified at the class-level using Resistome Analyzer (https://github.com/cdeanj/resistomeanalyzer).

**Statistical Analyses:** For resistome comparisons, we anticipated approximately 80% power to detect a 16% difference, or a 1.16-fold difference between treatment arms, in macrolide resistance determinants. The effect of azithromycin on resistance determinants was analyzed using the ratio of the antibiotic resistance determinants in the two arms. Specifically defined as the mean normalized read count of combined antibiotic resistance determinants classified at the class level in the azithromycin treated group divided by the corresponding mean quantity in the placebo group. The primary outcome was the ratio of macrolide resistance determinants at the 48-month visit. The ratios of macrolide resistance determinants at the 36-month visit and all other classes of resistance determinants at both visits were secondary analyses. A 95% permutation confidence interval for each effect size was estimated by assuming a multiplicative effect of azithromycin treatment on read counts^17^. All analyses were done using the R program v.3.6.2 for Linux (R Foundation for Statistical Computing, Vienna, Austria).

TML, JDK, and TD designed and supervised the study. TCP performed the randomization. AMA, RM, AA, C Cook, EL, KSO, CEO, JDK, and TML oversaw the field work and sample collection. TD, LZ, and C Chen performed laboratory related experiments. EDC assisted with sample sequencing. ML assisted with data interpretation. TD, AH, LW, TCP performed the bioinformatics analyses with contributions from ML and TML. TD and TML wrote the initial draft, and all coauthors reviewed the manuscript and agreed to publication. TD, AH, LW, TCP, JDK, and TML vouch for the data.

## RESULTS

**Figure 1:**
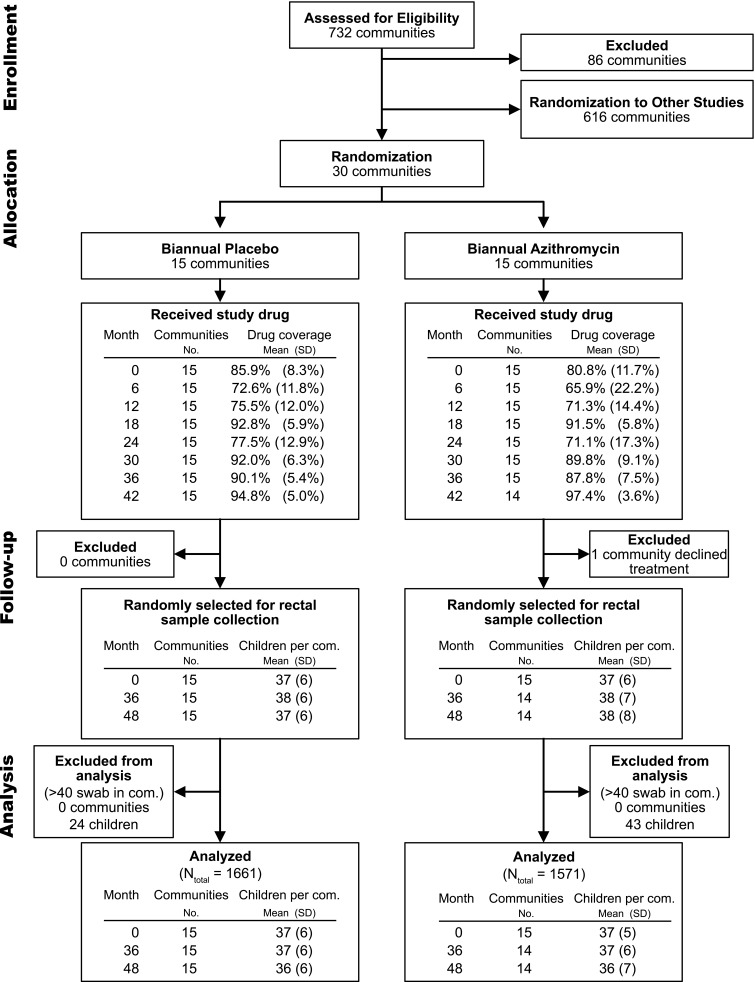
Study Profile

Thirty villages were randomized to biannual mass drug administration with oral azithromycin or placebo for 48 months. One village declined participation after the 24-month time point due to a combination of internal politics and study fatigue (Figure 1). Children aged 3-59 months in all communities in the study area received between 2 to 4 monthly distributions of seasonal malarial chemoprevention (SMC) with sulfadoxine, pyrimethamine, amodiaquine in the 2018 malaria season (July to August 2018, approximately 8 months prior to the 48-month collection). Study drug coverage over the eight biannual treatments was 83.2 ± 16.4% (± standard deviation) for azithromycin and 86.6 ± 12.0% for placebo. Across the baseline, 36 and 48-month visits, an average of 37 ± 6 children per village provided rectal samples. After imposing the 40-swab per village cap, a total of 3232 samples were processed, sequenced, and analyzed (1661 from placebo arm and 1571 from azithromycin arm) (Figure 1). Characteristics of participants contributing swabs are shown in Table 1.

**Table 1:** Demographics of Analyzed Participants

	*Rectal Swabs*					
	*Baseline*		*36 months*		*48 months*	
	Placebo	Azithromycin	Placebo	Azithromycin	Placebo	Azithromycin
Number of children	561	554	554	513	546	504
Mean age, months (95% CI)	31 (30 to 32)	31 (30 to 33)	30 (29 to 31)	31 (29 to 32)	32 (31 to 34)	31 (30 to 33)
Female, % (95% CI)	46 (42 to 50)	48 (43 to 53 )	46 (41 to 52)	45 (40 to 50)	47 (43 to 51)	45 (40 to 50)

**Figure 2:**
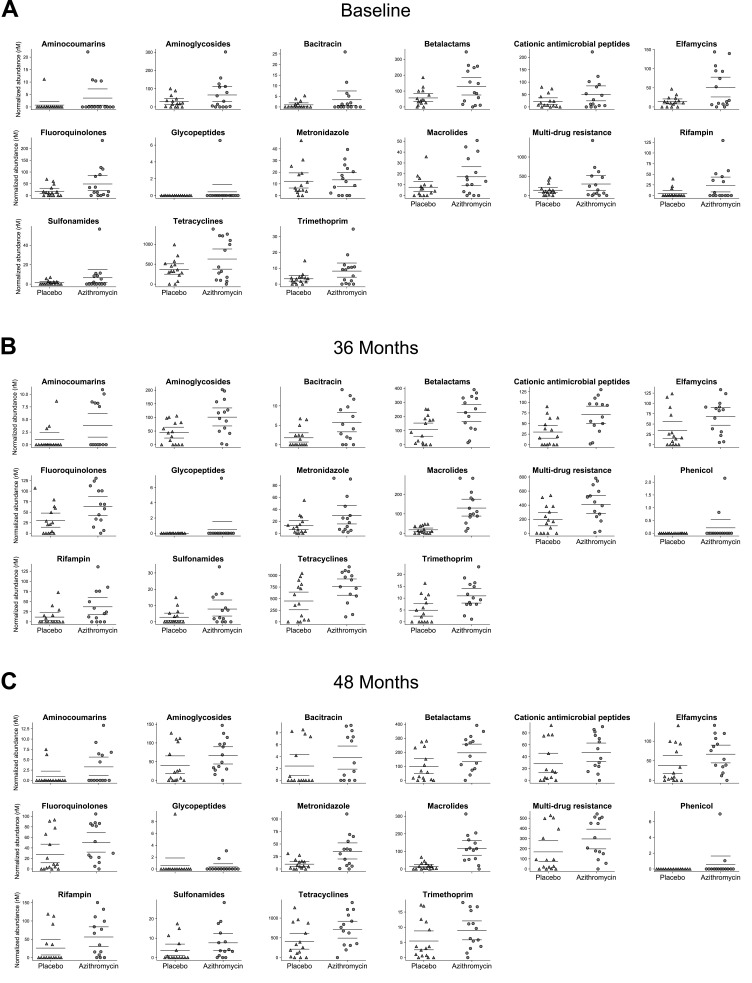
**Normalized antibiotic resistance determinants for placebo- and azithromycin-treated villages at baseline, 36, and 48 months.** Bars indicate the mean and 95% confidence intervals. Each point represents a village. “Multi-drug resistance” represents a class of genes that encode for low affinity efflux pumps. rM represents reads per million.

**Figure 3:**
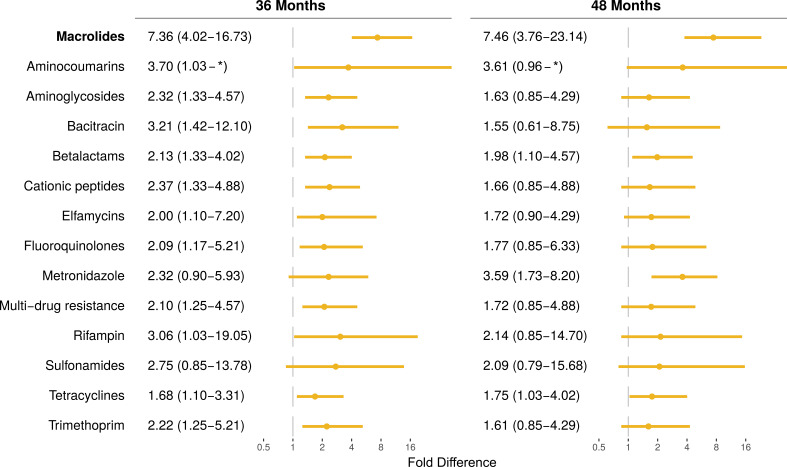
**Antibiotic resistance determinants in the gut of children aged 1-59 months after the 6^th^ and 8^th^ azithromycin distributions.** Fold difference of antibiotic resistance determinants in the azithromycin treated group compared to the placebo treated group with associated 95% confidence interval (95% CI). * indicates unbounded upper confidence interval.

At baseline, before any study treatments, the abundance of macrolide genetic resistance determinants were similar in the two treatment groups (Figure 2). At 36 months (i.e., after 6 biannual distributions), villages treated with azithromycin had a 7.4-fold greater abundance of macrolide resistance determinants than did communities treated with placebo (95% confidence interval 4.0 to 17.9-fold higher; Figures 2 and 3). These findings are consistent with the increase in macrolide-specific resistance detected after 4 distributions at earlier points of the trial^4^. In contrast to prior findings, an additional 2 rounds of mass azithromycin distribution caused a notable increase in resistance determinants to several other non-macrolide antibiotics (Figure 2 and 3), including a 2.1-fold greater abundance of beta-lactams resistance determinants (95%CI 1.2 to 4.0-fold). For the non-macrolide antibiotics that were elevated at 36 months, point estimates of the relative fold-difference at 48 months (after 8 distributions) were slightly lower, and in all cases not different from 1. An increase in macrolide resistance determinants persisted 6 months after the 8^th^ distribution (7.5-fold difference, 95% CI: 4.0 to 21.7-fold, Figure 3).

## DISCUSSION

We showed previously that two years of biannual mass azithromycin distributions in Niger resulted in an increase in macrolide resistance determinants in the gut^4,5^. As possibly expected, additional azithromycin distributions appeared to be associated with perpetuation of the increase in macrolide resistance, as seen here at the 36- and 48-month time points.

Until this study, we were unable to detect an increase in non-macrolide resistance with mass azithromycin distribution. Notable were the increases in resistance determinants identified in 4 antibiotic-classes (aminoglycosides, beta-lactams, trimethoprim, and metronidazole), each of which belongs to the World Health Organization’s ACCESS group of antibiotics given their effectiveness against a wide range of commonly encountered pathogens^18^. Of particular interest are genetic determinants of beta-lactams antibiotic resistance, as this class of antibiotics is widely utilized in sub-Saharan Africa^19^.

The increase of antibiotic resistance between the 4^th^ and 6^th^ distribution in the same communities is suggestive of a cumulative effect of azithromycin on the collective community gut microbiome. While azithromycin preferentially reduces susceptible pathogens, such as *Campylobacter* species, it may also affect the abundances of other species in the gut^5^. Thus, under the selection pressure of azithromycin, not only are gut bacteria harboring macrolide resistance determinants potentially selected for, but bacteria carrying non-macrolide resistance determinants may be sometimes favored, if they reside in the same bacterial lineages^20^. The selection of plasmid encoded resistance genes, such as the *erm* class methylated genes, also may have implications for horizontal gene transfer. Previous studies in other populations have shown associations between treatment with one drug class and rises in resistance to other drug classes ^21-24^. In general, co-occurrence of resistance mechanisms to different, unrelated drug classes is far more common than would be expected by chance alone^20,25^.

The potential implications for the increase of the community gut resistome with repeated mass azithromycin distribution are multifold. Resistant bacteria may mitigate the beneficial effects of azithromycin, although we have yet to observe that ^3^. Indeed, the efficacy of azithromycin in reducing childhood mortality actually increased as macrolide resistance was accumulating over the first two years of treatments in MORDOR I ^2,4^. From a public health standpoint, more concerning would be the potential for the propagation of non-macrolide and macrolide resistance genes to areas untreated with azithromycin. However, mass azithromycin distribution continues to be effective, despite the distribution of more than 860 million doses of azithromycin worldwide for the elimination of trachoma alone^26,27^. It remains the WHO’s recommendation for trachoma control ^28,29^. In addition, the prevalence of antibiotic resistance has been shown to predictably decline when mass drug distributions are discontinued, at least for certain antibiotics such as azithromycin^30,31^.

While we also detected some evidence of selection of non-macrolide resistance determinants, the difference between the azithromycin and placebo arms was more compelling at 36 months than at 48 months. For multiple drug class analyzed, however, point estimates or resistance were higher in the azithromycin-treated communities at both study visits. Although it is not clear how much genetic resistance determinants correlate with phenotypic resistance, the findings highlight the potential for broad antibiotic resistance even when a single antibiotic is repeatedly distributed in the community. Currently, health care providers in regions receiving mass azithromycin distribution for trachoma are alerted to the possibility of increased macrolide resistance. Any program that involves mass drug distribution for childhood mortality would need to inform providers and monitor for antimicrobial resistance. The increase of antibiotic resistance determinants across multiple antibiotic class observed in this study suggests that the routine practice of antibiotic resistance surveillance by performing phenotypic drug resistance profiles on any single model organism may be insufficient to provide a comprehensive understanding of the overall changes in antibiotic resistance in the community^32^. As metagenomic approaches become more routine, it may be useful to combine phenotypic and genomic approaches to monitor changes in antibiotic resistance.

Several limitations of the study should be noted. The storage of our rectal samples precludes phenotypic assessments of the gut organisms, preventing the direct identification of potential organisms that have increased non-macrolide resistance, and thus limiting mechanistic insights. ^4^ We did not collect data on symptoms of infectious illnesses in sampled children, nor on the occurrence of clinically resistant infections at local health posts, limiting the ability to make clinical inferences from the data. Here, we addressed colonization in a random sample of children, regardless of symptoms. Other studies will be necessary to document whether azithromycin distributions have increased resistance to macrolides and other antibiotics in symptomatic children who present to health posts or hospitals. The study region began to receive seasonal malarial chemoprevention prior to the 48 months visit. While this should not affect the relative fold-difference in resistance genes between treatment arms in a randomized trial setting as children in both arms received SMC, we cannot fully rule out potential confounders. Randomization was done at the village level, in which all children in a village were offered treatment, and thus the treatment adherence and the number of treatments cannot be interpreted at the individual level. Similarly, the outcome is a community average load of antimicrobial resistance genes, and therefore single individuals could disproportionately affect that average. Finally, the generalization of these findings to populations beyond similar rural settings of Niger should be done with caution.

In summary, this placebo-controlled, community-randomized trial showed that biannual mass azithromycin distributions for 4 years were associated with an increase of both macrolide and non-macrolide resistance genes. Resistance surveillance should be an intrinsic component of any mass drug distribution program and it can be achieved with metagenomic approaches.

Disclosure forms provided by the authors are available with the full text of this article at NEJM.org.
