# Supplementary material for: Selection of Macrolide and Non-Macrolide Resistance with Mass Azithromycin Distribution: A Community-Randomized Trial
Source: N Engl J Med. Author manuscript; Available in PMC 2020 Nov 12. (PMC7492079; doi:10.1056/NEJMoa2002606)
Supplement: Supplementary file 1 [file NEJM-2020-2002606-s1.pdf]

Table of Contents

Collaborators…………………………………………………………………2
Acknowledgements……………………………………………………….....2

Collaborators

The following investigators participated in MORDOR: *University of California, San Francisco, San Francisco, CA, USA* – Catherine A Cook, Sun Y Cotter, Thuy Doan, Dionna M Fry, Bruce D Gaynor, Jeremy D Keenan, Elodie Lebas, Thomas M Lietman, Kieran S O'Brien, Catherine E Oldenburg, Travis C Porco, Kathryn J Ray, Philip J Rosenthal, George W Rutherford, Nicole E Stoller, Benjamin Vanderschelden, John P Whitcher, Zhaoxia Zhou, Lina Zhong; *London School of Hygiene and Tropical Medicine, London, UK* – Robin L Bailey, Sarah E Burr, John Hart, David CW Mabey, Anthony W Solomon; *Johns Hopkins University, Baltimore, MD, USA* – Evan M Bloch, Christian L Coles, Kurt Dreger, Hemjot Kaur, Alain B Labrique, Beatriz Munoz, Alfred Sommer, Jerusha Weaver, Sheila K West; *Blantyre Institute for Community Ophthalmology, Blantyre, Malawi* – Alvin Chisambi, Khumbo Kalua, Zachariah Kamwendo; *University of Malawi College of Medicine, Blantyre, Malawi* – Ken Maleta; *The Carter Center, Atlanta, GA, USA* – E Kelly Callahan, Aisha E Stewart; *The Carter Center Niger, Niamey, Niger* – Ahmed M Arzika, Abdou Moumouni Goundara, Salissou Kane, Ramatou Maliki; *Programme National de Santé Oculaire, Niamey, Niger* – Amza Abdou, Nassirou Beido, Boubacar Kadri; *Muhimbili University of Health and Allied Sciences, Dar es Salaam, Tanzania* – Mabula Kasubi; *National Institute for Medical Research, Dar es Salaam, Tanzania* – Leonard Mboera, Zakayo Mrango; *International Trachoma Initiative, Decatur, GA, USA*- Paul M Emerson, Huub Gelderbloom, MD, PJ Hooper.

The steering committee for the trial consisted of the following investigators: Robin L Bailey, Jeremy D Keenan, Thomas M Lietman (PI), Travis C Porco, and Sheila K West.

Acknowledgements

We thank the program officers from the trial's sponsor: *Bill & Melinda Gates Foundation, Seattle, WA, USA*– Rasa Izadnegahdar, Julie Jacobson, Thomas Kanyok, Erin Shutes. We also thank the members of the Data and Safety Monitoring Committee: *University of Washington, Seattle, WA, USA* – Judd L Walson; *Liverpool School of Tropical Medicine, Liverpool, UK* – Allen W Hightower; *Loyola University, Chicago, IL, USA* – Emily E Anderson, *Berhan Public Health & Eye Care Consultancy, Addis Ababa, Ethiopia* – Wondu Alemayehu; *Tulane University, New Orleans, LA, USA* – Latha Rajan.
